# Catchment memory explains hydrological drought forecast performance

**DOI:** 10.1038/s41598-022-06553-5

**Published:** 2022-02-17

**Authors:** Samuel Jonson Sutanto, Henny A. J. Van Lanen

**Affiliations:** 1 Hydrology and Quantitative Water Management Group, Environmental Sciences Department, Wageningen University and Research, Wageningen, The Netherlands; 2 Water Systems and Global Change Group, Environmental Sciences Department, Wageningen University and Research, Droevendaalsesteeg 3, 6708 PB Wageningen, The Netherlands; 3 Institute for Marine and Atmospheric research Utrecht, Utrecht University, Princetonplein 5, 3584 CC Utrecht, The Netherlands

**Keywords:** Hydrology, Natural hazards

## Abstract

Hydrological drought forecasts outperform meteorological ones, which is anticipated coming from catchment memory. Yet, the importance of catchment memory in explaining hydrological drought forecast performance has not been studied. Here, we use the Baseflow Index (BFI) and the groundwater Recession Coefficient (gRC), which through the streamflow, give information on the catchment memory. Performance of streamflow drought forecasts was evaluated using the Brier Score (BS) for rivers across Europe. We found that BS is negatively correlated with BFI, meaning that rivers with high BFI (large memory) yield better drought prediction (low BS). A significant positive correlation between gRC and BS demonstrates that catchments slowly releasing groundwater to streams (low gRC), i.e. large memory, generates higher drought forecast performance. The higher performance of hydrological drought forecasts in catchments with relatively large memory (high BFI and low gRC) implies that Drought Early Warning Systems have more potential to be implemented there and will appear to be more useful.

Skillful Drought Early Warning Systems (DEWSs) to predict drought a few months in advance are of utmost importance to reduce the impacts of the drought hazard^1,2^. Previous studies on meteorological drought forecasts using the Standardized Precipitation Index (SPI)^3^ show that (1) drought can be sufficiently predicted up to 1–3 months ahead, depending on the accumulation period x (e.g., SPI-x, with x = 1, 3, 6, or 12 months) and (2) the highest drought forecast skill is obtained for SPI with longer accumulation periods^4–7^. The monthly temporal resolution used in the seasonal drought forecasts, e.g. SPI that measures drought by the deviation of monthly precipitation values from long-term median precipitation for each month^8,9^, is the main cause that escalates the weather forecast skill^10^, which commonly (in terms of below normal precipitation) has skill only for a few days up to a few weeks^11,12^. Drought forecasts, however, do not require detailed day-to-day evolution, as demanded in conventional weather forecasts. Instead, drought predictions, such as SPI, need estimates of monthly total precipitation, that provide information on how likely the coming months will be wetter or drier than the median^13^. For example, over- or underestimation of precipitation by 10 mm on a daily basis is crucial for weather forecasting in general and flood forecasting in particular. For drought forecasting, missing or having more precipitation of 10 mm on a certain day in a month is less significant as long as the predicted monthly median of precipitation derived from the ensembles is fairly reliable, i.e. it can be counteracted by more or less precipitation on other days in the month. In addition to the monthly accumulation period, the blending of preceding monthly-observed data with forecasts to calculate drought indices with higher accumulation periods (e.g. SPI-x, x > 1) for short lead times (LT, e.g. LT = 1-month) mainly explains meteorological drought forecast skill^4,7,14–16^.

The skill of hydrological drought forecasts, on the other hands, is even 2–3 months higher than the meteorological ones^7,16^. Sutanto et al.^7^ show that hydrological drought forecasts with 1-month accumulation period identified using the Standardized Runoff Index (SRI-1)^17^ and Standardized Groundwater Index (SGI-1)^18^ and LT = 1 have perfect forecasts up to 71.5% and 73.2% of the pan-European area, respectively, which is higher than meteorological drought scores (53.7% for SPI). Another study by Van Hateren et al.^16^ supports this finding. They show that streamflow drought forecasts outperform climatology up to LT = 4-month while the skill of meteorological forecasts (SPI-1 and SPI-3) is higher than climatology only for LT = 1-month. In addition to the accumulation period and the blending of observed and forecasted data (see above), the high skill in hydrological drought forecasts is anticipated coming from the land surface water storage/memory (e.g. lakes, soils, groundwater) that pools, attenuates, and lengthens the effect of the driving forces (i.e. precipitation)^19^. Recent studies by Pechlivanidis et al.^20^ and Girons et al.^21^ on seasonal streamflow forecasts (not streamflow drought) also conclude that the predictability is higher in the slowly responding basin, associated with large catchment memory. The effect of catchment memory on hydrological drought forecasting performance, however, has never been revealed yet.

Here, we have conducted a pioneering study that investigates the importance of catchment memory on the forecast performance of streamflow drought across Europe. Please note that streamflow drought forecasts deviate from streamflow forecasts (or generally hydrological forecasts). Drought forecasting requires an additional step using the forecasted time series of a hydrological variable, that is, forecasted drought is a derived product from hydrological forecasts. The flow time series need to be converted into a time series of drought events by applying drought identification approaches, e.g. the threshold approach or the standardized approach. In this study, we identify streamflow drought using the Standardized Streamflow Index^22^ for different subregions in Europe^23^. The observed and forecasted streamflow droughts at major European rivers were derived from the streamflow data obtained from the European Flood Alert System (EFAS) driven by observed and forecasted weather data^24,25^. Catchment memory was derived from: (1) the Baseflow Index (BFI)^26^ that explains the capacity of a catchment to store and release water from catchment sources of water, and (2) the groundwater Recession Coefficient (gRC) that describes storage and releases of water from subsurface water. The forecast performance was evaluated using the Brier Score (BS) approach^27^ and correlated with BFI and gRC (“Methods”). Our results show that the highest forecast performance is found in rivers with higher BFI and lower gRC values, associated with higher memory, which explain the importance of considering catchment memory in improving the performance of hydrological drought forecasts.

## Results

### The Baseflow Index (BFI) for major European rivers

The BFI is commonly used to indicate the portion of the flow, i.e. baseflow, that comes from groundwater storage or other delayed sources, which is derived from recessions in streamflow^26,28,29^ (“Methods”). Rivers with a flashy flow regime, associated with small catchment memory due to, e.g. less permeable soil layers, no or limited aquifer storage, force rainfall to flow quickly to the stream, have typically low BFI values^30^. Vice versa, rivers in catchments with large memory yield high BFI values. The rivers located in mountain ranges or snow-dominated regions generate high BFI (Supplementary Fig. 1) that may come from water stored and released from snow and ice and the presence of lakes and wetlands^20,31^. The BFI of rivers in other European regions ranges from 0.1 to 0.8. In these regions, BFI is predominantly controlled by other catchment properties. For example, a big difference in BFI values is seen for some rivers flowing in France and the UK both located in Western Europe. Rivers flowing in France have BFI values between 0.3 in the upstream and 0.7 in the downstream, while rivers in the UK have lower BFI values range from 0.1 to 0.6 (Supplementary Fig. 1). The aquifer types in these countries explain the different BFI values. North and west UK have little catchment storage compared to France, where major aquifer system occur in some areas^32,33^.

Supplementary Figure 2b–g show examples of BFI varying from 0.78 to 0.19 for six rivers located in different European subregions^23^, with at least one river represented in each region. The Danube and the Rhine Rivers have the highest BFI values (0.78 and 0.74, respectively). These rivers are identified as baseflow dominated rivers, which have long recessions and small response to precipitation^20^. The annual climatic water balance (precipitation minus evapotranspiration) in these rivers is around 200 mm with a runoff coefficient of 0.45 (Supplementary Table 1). The Guadiana and the Tanaro Rivers located in the southern European region yield lower baseflow than the Danube and Rhine located in central Europe with BFI values of 0.66 and 0.51, respectively. These rivers, especially Guadiana, have lower precipitation and higher evapotranspiration than the Rhine and Danube Rivers. Although the Tanaro River has similar average baseflow to the Guadiana, it is flashier in the high flow range, resulting in a lower BFI (Supplementary Fig. 2d,e). The Tweed and Kolbacksan Rivers experience the lowest BFI values due to low baseflow and quick response to precipitation. These rivers situate in regions that are characterized by limited aquifer storage^34,35^ and hence have low catchment memory and fast catchment response, with a runoff coefficient up to 0.62 for the Tweed River (Supplementary Table 1).

### Drought forecast performance for major European rivers and connection with BFI

**Figure 1:**
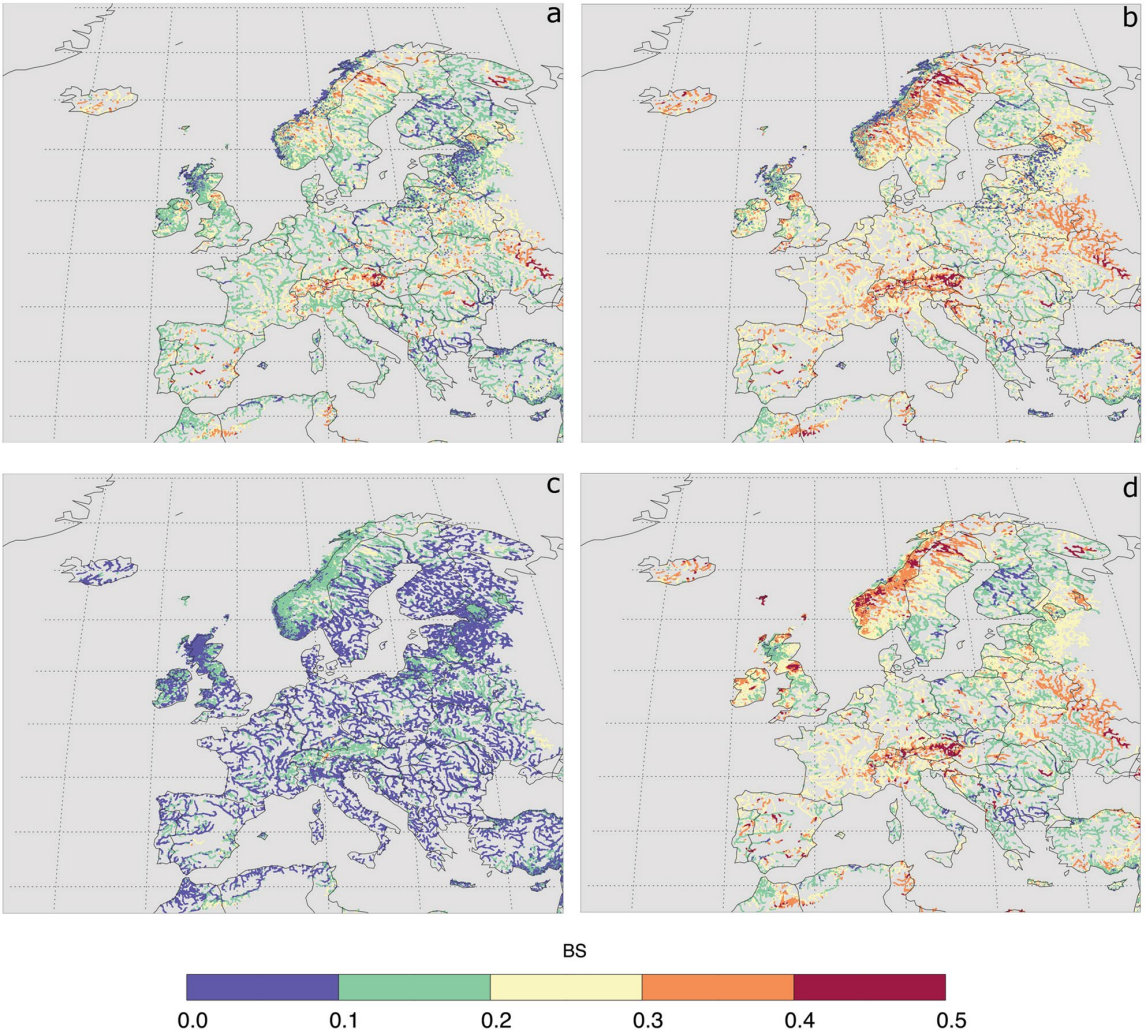
Drought forecast performance denoted by the Brier Score (BS) for major European rivers: (**a**) BS for SSI-1 with Lead Time (LT) = 1-month, (**b**) BS for SSI-1 with LT = 3-month, (**c**) BS for SSI-3 with LT = 1-month, and (**d**) BS for SSI-3 with LT = 3-month. Note all figures were plotted using the Interactive Data Language (IDL) software version 8.0 (https://www.l3harrisgeospatial.com/Software-Technology/IDL).

Using the re-forecast and proxy observed data (SFO, see “Methods”), the drought forecast performance for major European rivers was calculated using the Standardized Streamflow Index with accumulation periods of 1 month (SSI-1)^22^ and of 3 months (SSI-3) with lead times (LTs) of 1 and 3-month (Fig. 1). The performance of drought forecasts was determined by using the Brier Score (BS), which indicates a higher performance when BS is low^27^ (“Methods”). The highest performance is obtained in some rivers located in Finland, Hungary, Poland, north Germany, and east Romania (Fig. 1a,b,d). Rivers, situated in the mountainous regions, such as in the Alps and east Norway, have the lowest performance, although these rivers have fairly high BFI (Supplementary Fig. 1). The low performance of drought forecasts in these regions relates to the timing of snow occurrences. The mismatch in the prediction of too early or late snow accumulation or melt is caused by the mismatch in the prediction of temperature and precipitation, resulting in a bias in the temperature-dependent simulation of snowfall and snowmelt^7,36,37^. For example, the prediction of too high temperature leads to early snowmelt. On the other hand, the prediction of too low temperature will result in more snow accumulation, which otherwise would have resulted in rainfall infiltrating into the soil. Moreover, the meteorological forecast data used as forcing in the EFAS are not bias corrected (ECMWF, personal communication). SSI-3 with 1-month LT attains the highest performance due to the blending of 2 months observed (SFO) data with one month forecast (Fig. 1c). The performance deteriorates with an increased of LTs (Fig. 1a vs. b and c vs. d).

**Figure 2:**
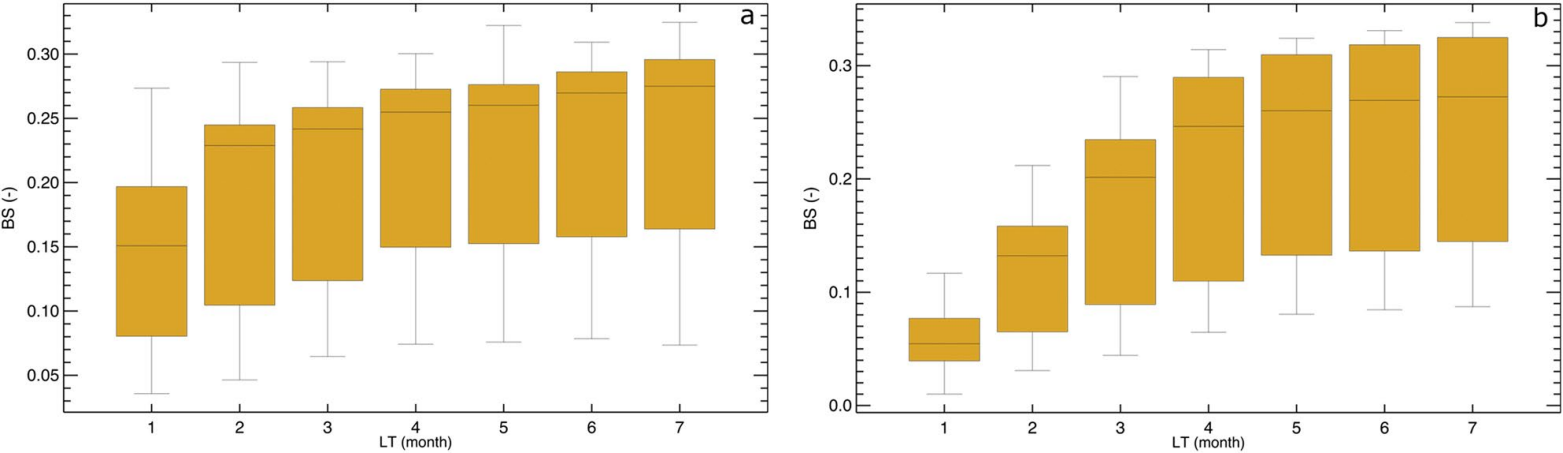
Box and whisker plot showing drought forecast performance denoted by the Brier Score (BS) for major European rivers: (**a**) BS for SSI-1 with LTs from 1- to 7-month and (**b**) BS for SSI-3 with LTs from 1- to 7-month. Lower box shows 25 percentile, middle line shows median, and upper box shows 75 percentile. The whiskers show the 10 and 90 percentiles.

A summary of SSI-1 and SSI-3 drought forecast performance for major European rivers and for all LTs is presented in Fig. 2. A sharp decrease in forecast performance (increase of BS) is clearly seen from LT = 1 to LT = 3. For LTs beyond 3-month, the forecast performance does not significantly change, meaning that these drought forecasts do not provide added value, as it was also found in previous studies^6,7,16^. Both SSI-1 and SSI-3 with LT > 3 show similar forecast performance (BS = 0.24–0.26 for ensemble median). As expected, the highest performance is obtained for SSI-3 due to the blending of observed data with forecasts (Fig. 2b). Furthermore, the performance of drought forecasts varies over seasons, depending on the accumulation period and LT. The highest performance derived from the median ensemble is obtained for SSI-3 with LT = 1 in summer, followed by spring, autumn, and winter seasons (BS < 0.07, Supplementary Fig. 3). For SSI-1 with LT = 1, the highest performance is obtained in summer followed by autumn, winter, and spring seasons (BS < 0.15). For the longer lead time (LT = 3), the highest/lowest performance is obtained in winter/autumn and spring/autumn for SSI-1 and SSI-3, respectively. In general, the drought forecasts perform well in summer for LT = 1 and in winter and spring for LT = 3.

**Figure 3:**
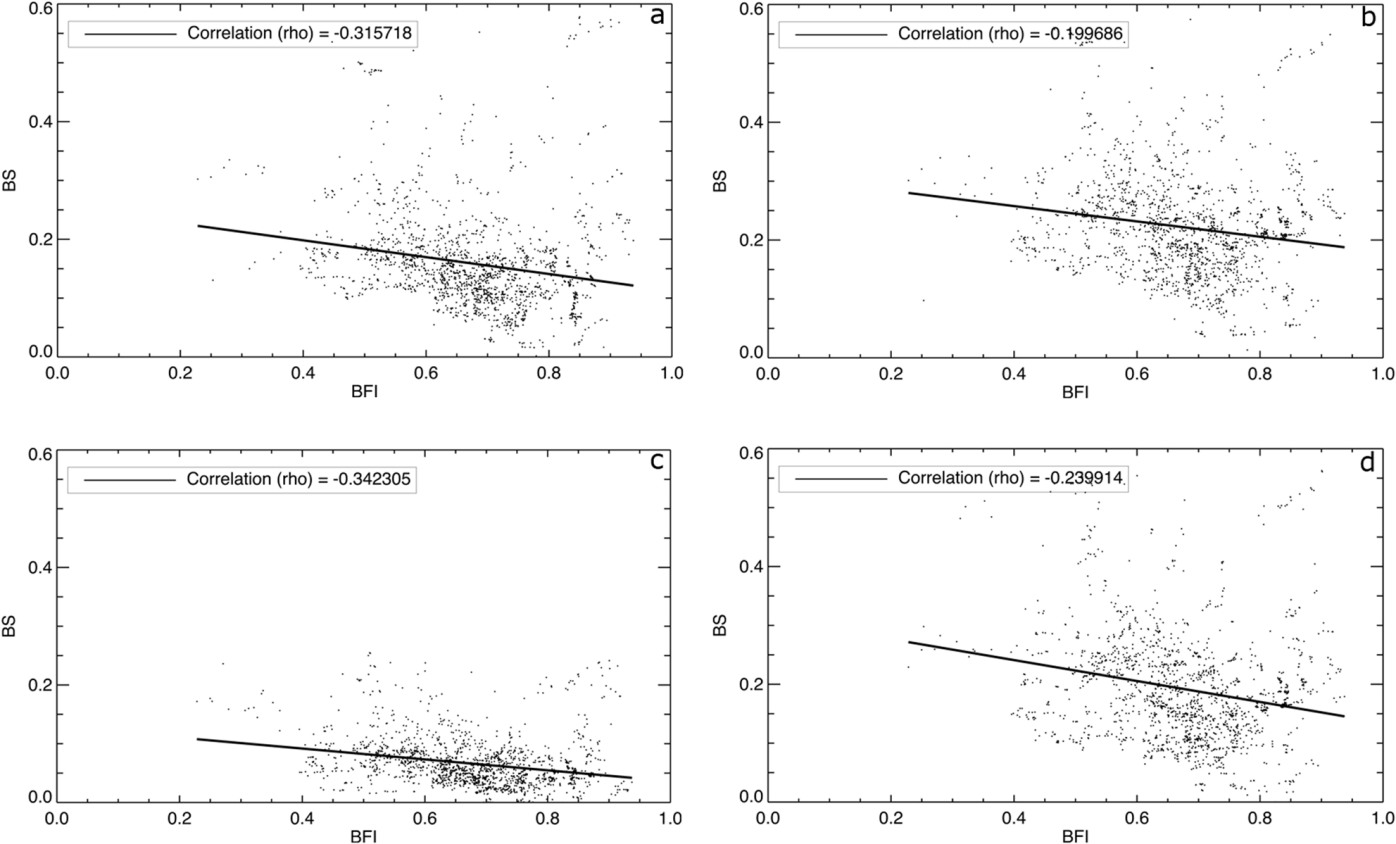
Correlation between BFI and BS for southern European river grid cells: (**a**) for SSI-1 LT = 1, (**b**) for SSI-1 LT = 3, (**c**) for SSI-3 LT = 1, and (**d**) for SSI-3 LT = 3. See Supplementary Table 2 for average BFI and BS values in each European subregion and Europe as a whole.

The hydrological persistence of a river with high BFI causes high streamflow forecast performance^20,21,38^. A high BFI means that there is a rather high probability that streamflow in the next days will not be too much different from today’s streamflow. Likely, this also applies to the dry anomalies in streamflow (i.e. drought), that is, if the river is below normal today, then it is also expected to be below normal in the days after. However, the persistence in streamflow drought is observed^39^, but not well understood yet. To prove our hypothesis that the higher performance of drought forecasts is obtained for rivers with higher BFI, we plotted the correlation between BFI and BS for all major river grid cells (n = 10,106) in southern Europe (Fig. 3 and Supplementary Table 2 for other European regions). Overall, significant negative correlations (p < 0.05) are obtained with higher Spearman’s correlation values (Rho) for LT = 1 than LT = 3, as expected (Fig. 3a vs. b and c vs. d). Lower forecast performance for LT = 3 is illustrated by more spread in BS and lower Rho, particularly for SSI-1. Compared to SSI-1 LT = 1, the BS values for SSI-3 LT = 1 are less scattered, associated with higher forecast performance for SSI-3 than SSI-1. The blending of observed data for drought standardized indices (e.g., SPI, SSI, and SRI) with an accumulation period of more than one month enhances the forecast performance, in particular for meteorological drought forecasts^4–7,15,16^. On average, the highest BFI values (0.71) and lowest BS (0.16) are found in rivers located in Central and South Europe, respectively (Supplementary Table 2), which indicate a potential for the development of drought early warning systems for rivers located in these regions. Conversely, rivers in West Europe have the lowest BFI (0.61), but medium forecast performance (BS = 0.19). The lowest forecast performance is found in rivers located in North Europe (BS = 0.21).

Some past studies correlated the streamflow forecast skill with BFI (e.g. Harrigan et al.^38^ and Girons et al.^21^). They found that the forecast skill is high in catchments with high BFI. Our study on streamflow drought forecast performance supports their findings but with lower correlation coefficients. The somewhat lower correlation obtained in our study might be caused by that, in streamflow forecasting, the skill assessment is performed for all flow conditions, which include normal, high, and low flows. In streamflow drought forecasting, however, we evaluate the forecast performance only for extreme below normal events, i.e. anomalies called drought. The focus on only dry extremes is hypothesized to reduce forecast performance. Moreover, there are more indicators that can describe the catchment memory, such as hydrogeology, the relative area of lakes/wetland/bogs, topography, land use, and soil types that might affect correlation strength. Although the effects of these indicators are already to some extent embedded in the BFI, the use of more indicators than BFI may improve the correlation coefficient.

### The impact of groundwater memory

In previous sections, the BFI is used as the first metric to investigate the impact of catchment memory. In this section, we use the groundwater Recession Coefficient (gRC, Supplementary Fig. 4a) that more specifically signifies groundwater, as a second metric to further study the impact of catchment memory on drought forecast performance^40^. The performance was investigated by evaluating the relationship between groundwater gRC and BS (“Methods”). Here we consider 16 river catchments that are located in different European subregions (Supplementary Fig. 2a). These rivers have a broad range of BS and gRC values to derive the correlation. Unlike BFI that can be derived for all individual river cells, the gRC is investigated for river grid cells at the outlet, which is obtained by spatially averaging gRCs of all land cells upstream of the outlet (“Methods”) that contribute to groundwater flow (catchment, Supplementary Fig. 4b). The time-consuming spatial averaging is the main reason that estimating the correlation between gRC and BS and between gRC and BFI is not feasible for all river grid cells across Europe (“Methods”). Hence, we selected several catchments that reflect a broad range of physio-geographic conditions across Europe. We infer that slowly responding catchments (large groundwater memory) denoted by low gRC will generate high drought forecast performance as it was proved for streamflow forecast.

**Figure 4:**
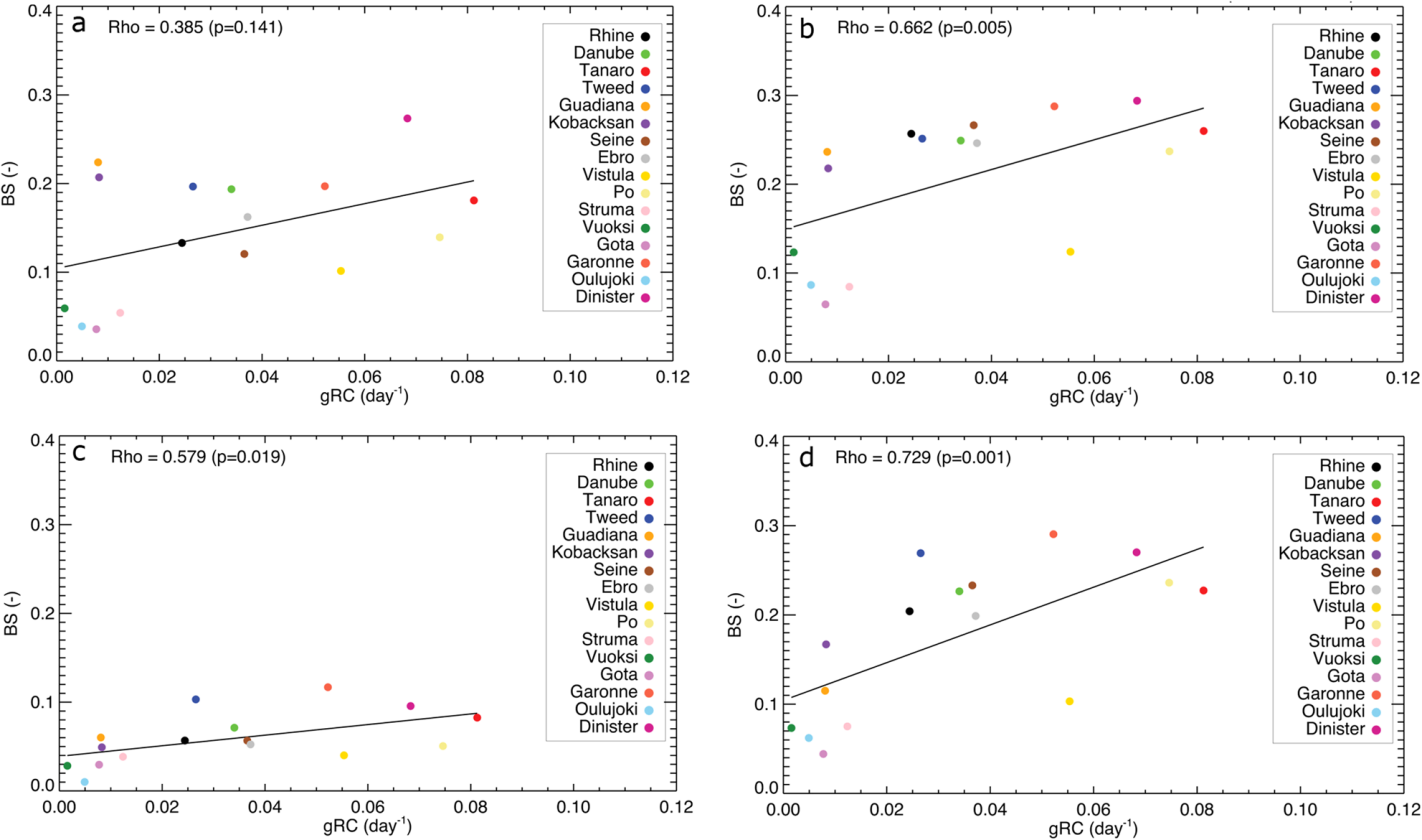
Correlation between BS and gRC for the 16 selected river catchments: (**a**) for SSI-1 LT = 1, (**b**) for SSI-1 LT = 3, (**c**) for SSI-3 LT = 1, and (**d**) for SSI-3 LT = 3.

Figure 4 shows that catchment with lower gRC associated with a slow release of groundwater from the system produces higher drought forecast performance. Strong positive correlations between gRC and BS are seen for both SSI-1 and SSI-3 and for all LTs (Rho > 0.5, except for SSI-1 LT = 1). The correlation, however, becomes more robust (increase up to > 0.7) for SSI-3 (Fig. 4c,d) than SSI-1 (Fig. 4a,b), because two months preceded observation data are included. Rivers located in northern Europe, such as Vuoksi, Göta, and Oulujoki, have low gRC and high forecast performance. This might be due to the presence of lakes in their catchments that attenuate streamflow peaks owing to storage and evaporation^41^. Rivers located in the mid latitudes (Danube, Vistula, Rhine, Tweed, Seine, Dinister, and Garonne) in general have similar forecast performance and gRC, with higher performance relatively found in the rivers in east Europe. Mediterranean rivers (Struma, Guadiana, Ebro, Po, and Tanaro) have a wide range of gRCs from 0.01 to 0.08 and BS from 0.06 to 0.22 (Fig. 4a). The Guadiana River in Spain and the Struma river in Greece that have slow catchment response (gRC = 0.008 and 0.012, respectively) yield different prediction performance (BS = 0.22 and 0.05, respectively, Fig. 4a). Anthropogenic activities to cope with water scarcity in the Mediterranean region, such as the rapid development of reservoirs in the past decades, modified the flow regime^42^. A clear example is seen in the Guadiana River, which has 39 man-made reservoirs located along the catchment^43^. It seems that the reservoir regulation and management highly influence the flow regime and associated hydrological drought^44^ in this river and thus reduce the forecast performance.

We also tested our hypothesis that catchment with lower gRC generates higher drought forecast performance using a dichotomous forecast method for comparison. The forecast performance is identified based on forecast accuracy and success ratio (SR), which is 1 for perfect forecast (see Supplementary Note). Supplementary Figure 5 shows negative correlations between gRC and forecast accuracy, as well as forecast SR (Rho $< -$ 0.5), which support our hypothesis using BS. Note that the correlation sign of accuracy and SR is opposite to the correlation using BS because higher forecast performance is reflected by higher accuracy and SR, unlike BS that shows lower performance for higher BS. On average, catchments that have gRC values lower than 0.04 (high memory) have higher prediction performance, with more than 80% of all forecasts that were correct (accuracy > 0.8, Supplementary Fig. 5a) and 70% of the forecasted drought events were actually observed (SR > 0.7, Supplementary Fig. 5c).

The relation between gRC and BFI is also provided in Supplementary Fig. 6. The correlation between gRC and BFI is negative. This is not surprising because catchments with high gRC are associated with a quick release of groundwater and fast responding streamflow (low BFI). The correlation (Rho = − 0.376), however, is rather weak and not significant at p < 0.05. The weak correlation found in our study might be caused by the small number of river catchments (16 catchments) to confirm the dependency. Using 314 catchments in the UK, Harrigan et al.^38^ found a strong correlation between BFI and catchment storages (soil moisture and groundwater), with a correlation coefficient up to 0.87. Although the BFI in many applications is used as a measure of streamflow that originates from the subsurface storage (e.g., groundwater), it also measures catchment characteristics, such as lake area, wetland area, land cover, catchment slope, and catchment shape and area, which result in different streamflow responses to climate i.e., precipitation and evaporation^45,46^. Our study proved that groundwater memory (fast to slow release of groundwater), denoted by gRC, has a strong impact on drought forecast performance, in addition to other performance provided by other sources of water in the catchment, which are incorporated in the BFI.

## Discussion and conclusions

Our study shows that catchment memory is a key component in the predictability of hydrological drought, since it strongly influences hydrological drought characteristics^19,40,45^. Catchment memory pools, attenuates, and lengthens anomalies in driving forces i.e. precipitation.

The use of higher accumulation periods in hydrometeorological drought forecasts improves forecast performance. This is also shown in our study, where the SSI with 3 months accumulation period and lead time of 1-month (SSI-3, LT = 1) yields higher forecast performance than the SSI-1 for the same lead time, as shown in our study. However, multiple studies have indicated that hydrological drought forecasts that are not blended with observed data (e.g. SSI-1 and SRI-1) outperform meteorological drought forecasts (e.g. SPI-1) up to a few months ahead due to the non-linear physical processes in the catchment system that pool, attenuate, and lengthen the effect of the driving forces i.e. precipitation^7,16^. The performance is even higher for catchment that has large memory, as it is shown in our paper. The catchment memory as the key component increases the predictability of the hydrological drought.

Our study on streamflow drought forecasting across Europe confirms that hydrological drought forecast performance is higher in a river draining in a slowly responding catchment, associated with larger memory or storage in groundwater (high BFI, low gRC). We also highlight the importance of other water bodies that store water, such as lakes that reduce temporal streamflow variability in describing the high performance of streamflow drought forecasts. The significant negative correlation between BFI and BS proves that high forecast performance can be obtained for rivers with high BFIs. This conclusion is further supported by a correlation analysis of gRC and BS. A positive correlation between gRC and BS for 16 river catchments across Europe provides clear evidence (Rho > 0.4) that the rate of release of groundwater storage to the river is an important factor in the predictive performance. We further explored the effect of catchment size on forecast performance by dividing the catchments into small and medium-sized catchments (7 catchments) and large catchments (9 catchments). This investigation confirms the conclusion that high streamflow drought forecast performance (low BS) comes hand in hand with rivers having high catchment memory (high BFI and low gRC), irrespective of catchment size (Supplementary Fig. 7).

The blending of observed data with forecasts, e.g. SSI-3 for short lead times (LT < 2 months), further improves the correlation between gRC and BS (Rho > 0.6). One should note that the use of longer accumulation periods (x > 1 month) in hydrological drought forecasting should be done with caution. Hydrological variables, e.g. streamflow already comprise some catchment memory aspects (delayed flow from groundwater). Hence some studies recommend to use only 1 month accumulation period for hydrological drought analysis^18,47^. On the other hand, we need to realize that anomalies in the accumulated flow over a longer period (e.g. SSI-3) have relevance for some purposes, such as the management of surface water reservoirs.

Among different seasons, streamflow drought forecasts perform well in summer for LT = 1 and in winter/spring for LT = 3. Our finding that streamflow drought forecast has high performance in summer supports the study by Sutanto et al.^7^ on hydrological drought forecasts that the skill is higher in summer. However, some studies on streamflow forecast (note: not on streamflow drought) contradict with our findings (e.g., Crochemore et al.^48^; Bell et al.^49^; Arnal et al.^50^). They concluded that the skill of streamflow prediction is higher in winter than in summer. We argue that the results obtained from streamflow drought forecasts may differ from streamflow forecasts. In streamflow drought forecasts, the skill of forecast is determined by the ability to predict low streamflow (here SSI $< -$ 0.5), whereas the skill of streamflow forecasts is derived from the full flow spectrum from low to high flow. The moderate skill of winter hydrological drought forecasts might be linked to large-scale teleconnection patterns that affect European climate in winter. Some studies show that the North Atlantic Oscillation (NAO), the occurrence of blocking, and the North Atlantic jet stream are conjectured as strong predictors in winter meteorological forecasts in Europe^33,51,52^. The high skill in prediction of winter precipitation and temperature is then translated to better prediction of streamflow and subsequently streamflow drought. However, the skill of meteorological forecasts is limited for long-term predictions (> 15 days) due to the chaotic nature of the atmosphere^53,54^.

In this study, we used the large-scale hydrological model, LISFLOOD, with a spatial resolution of 5 km by 5 km. This implies that results were derived purely based on the large-scale model simulation. A model-based assessment may not fully replicate the observational-based assessment, especially for the extreme cases, such as drought, because of uncertainties of different modeling components; in particular, the sub-surface characteristics that are not specifically captured in large-scale continental-based modeling assessment^21^. The errors will be higher in the catchments that had no streamflow observations for model calibration and validation. Nevertheless, the LISFLOOD model was calibrated using 717 stations across Europe^55^, which means that a reasonable number of catchments was covered. Although the uncertainties in modeling and forecasting streamflow drought at a continental scale, i.e. Europe, are expected to be higher than at catchment scale, the use of a large-scale model provides indicative results of drought forecasts for all major European rivers. This will fill the gap in providing seasonal drought forecasts in regions that have no DEWS, especially in regions with slowly responding hydrological systems that have high catchment memory.

Given drought hazard and its impacts are projected to increase under a future warmer climate in multiple regions across the globe^56–58^, the development of DEWSs that also provides hydrological drought forecasts is highly recommended because they have higher skill than meteorological drought forecasts and they better serve different users managing water resources^59^. Our results suggest that the performance of the hydrological drought forecast module of a DEWS would be higher for rivers draining catchments in East Europe, followed by rivers in South, Central, and North Europe (Supplementary Table 2). On the contrary, rivers situated in West Europe have the lowest BFI and highest BS due to the relatively fast response from the groundwater storage (high gRC, Supplementary Table 3) that explains the low predictability of hydrological drought.

## Methods

### Data

Proxy of daily observed streamflow data from 1990 to 2018 was simulated using the LISFLOOD hydrological model fed by gridded meteorological observation data (EFAS, 5 by 5 km) collected from > 5000 ground observations (hereafter called Simulation Forced by Observations, SFO or proxy observed). The same model was run with the re-forecast (known as hindcast) meteorological data obtained from the European Centre for Medium-Range Weather Forecasts (ECMWF) SEAS5 to simulate the re-forecast streamflow data from 2002 to 2016 (180 months). This period covers major drought events in Europe, e.g. west and central Europe in 2003, south Europe from 2006–2008, east Europe and Russia in 2011, and central and east Europe in 2015. The seasonal re-forecasts of streamflow have a lead-time of 215 days (circa 7 months) and consist of 25 ensemble members. The streamflow data, both SFO and re-forecast, were provided by the ECMWF as part of the European Flood Awareness System (EFAS system version 3)^24,25,60^. In LISFLOOD, the potential evapotranspiration was calculated using the Penman–Monteith equation through the offline LISVAP pre-processor^61^. The soil moisture flux out of the subsoil and between the upper and lower soil layer are defined based on the Darcy’s law. The groundwater storage and flow are modeled using two parallel linear reservoirs following to the approached used in the HBV-96 model. The groundwater upper zone represents a quick runoff component, including fast groundwater and subsurface flow through macro-pores in the soil. Whereas, the lower zone represents the slow groundwater component that generates the baseflow. Surface runoff and flow from the two groundwater zones (5 $\times$ 5 km grid cells) are routed to the river network. A kinematic wave approach was used for routing the water movement in the river network ($\sim$ 10,106 river grid cells). The LISFLOOD model used in our study incorporates water abstraction for irrigation, water demands for the livestock, energy production and cooling, and manufacturing industry, and 1454 reservoirs across Europe^55^. The LISFLOOD model was calibrated by Arnal et al.^55^ using over 700 streamflow observation data across Europe, which results in a Kling-Gupta Efficiency (KGE) of > 0.75 for more than 42% stations, KGE between 0.5 and 0.75 for 33% stations, and only 25% of stations show KGE below 0.54. LISFLOOD proved to be able to adequately simulate streamflow and hence the modelled results can mirror observed spatial and temporal streamflow patterns across Europe. Detailed information on SFO and re-forecasts data taken from EFAS are provided by Sutanto et al.^1^ and Arnal et al.^50,55^. All data and figures were processed using the Interactive Data Language (IDL) software version 8.0.

### Standardized Streamflow Index (SSI)

The Standardized Streamflow Index (SSI) identifies the severity of the drought in the river^22,62^. The SSI indicates the degree of dryness by providing the streamflow deviation (anomaly) from the long-term median. The gamma distribution was applied to monthly streamflow data taken from the 1990–2018 records, which then is transformed into 12 normal distributions for each accumulation period and every month of the year. In this study, SSI-x was calculated for the accumulation periods of 1 and 3 months (SSI-1 and SSI-3, respectively). Then, these distribution parameters were used as a basis to identify drought events in the observed (1990–2018) and re-forecast time series (2002–2016). The gamma distribution was applied to all major European rivers since it is applicable for streamflow forecasting as shown in a previous study^63^. We would like to note that the gamma distribution is typically used to model highly skewed time series and it is not fully able to fit data at the lower tail (low streamflow in this study), leading to underestimation of drought^64^. Furthermore, the use of the gamma distribution in rivers that encounter zero flow conditions, e.g. in semi-arid and arid regions (not in this study), also needs attention because the gamma distribution cannot model zero streamflow. Other distributions for streamflow, such as log normal, Generalized Extreme Value (GEV), Tweedie, and nonparametric method can be considered^47,64^. However, studies done by Vicente-Serrano et al.^62^ and Tijdeman et al.^47^ show that none of the single distributions fits all streamflow time series across Europe and will fit well with all monthly streamflow data in all river grid cells, e.g., sample properties of streamflow in January likely will differ from those in August. Thus, we simply used the widely selected gamma distribution in our analysis.

The re-forecasted SSI-3 with a lead-time (LT) of k-month (k from 1 to 7-month) combines for LT $\le$ 2 month re-forecast data and observation data from preceding months (SFO streamflow), and uses for LT > 2 month only forecast data. For example, the re-forecasted SSI-3 for January 2003 with 1-month LT (forecast issued on 2nd January 2003) was estimated by combining the observed data from November and December 2002 with January 2003 re-forecast data (LT = 1). To re-forecast the SSI-3 for February 2003 with LT = 2 (forecast issued on 2nd January 2003), we combined the observed data of December 2002 with re-forecast data for January and February 2003. Thus, the re-forecasted SSI-3 with LT = 3 consists of only re-forecast data. Using SSI, a drought event was classified into four classes, which are: (i) mild drought: 0 > SSI $\ge -$1, (ii) moderate drought: −1 > SSI $\ge -$ 1.5, (iii) severe drought: − 1.5 > SSI $\ge -$ 2, and (iv) extreme drought: SSI $< -$ 2.

### Baseflow Index (BFI)

The BFI is considered a measure of streamflow that originates from stored sources of water in a catchment e.g. groundwater, lakes, bogs, glacier, and snow. It can also be used for indexing the effect of geology, land use, and catchment area on streamflow, which results in different streamflow responses^45,46^. Thus, the effects of geology, land use, and other catchment characteristics are implicitly considered in the BFI. The BFI has been used in many studies to indicate catchment memory, i.e. storage and release properties of catchments, incl. groundwater^30,31,38,45,46^. These studies show that catchments with high BFI values have large (groundwater) storage and slow release of water from other delayed sources (e.g. water stored in lakes, bogs, glaciers, and snow). A slowly responding catchment is associated with a high memory catchment. The opposite holds for a catchment with low BFI, which is more responsive to rainfall. The BFI is obtained by applying hydrograph separation and is calculated as:

1 $$\begin{aligned} BFI=\frac{V_{base}}{V_{total}}, \end{aligned}$$

where $V_{base}$ is the volume of water beneath the baseflow part of the hydrograph between the first and last date of interest. $V_{total}$ is the volume of water beneath the recorded hydrograph. Hisdal et al.^30^ provides a detailed procedure for BFI calculation. Examples of streamflow hydrograph and its baseflow for several selected rivers are presented in Supplementary Fig. 2b-g.

### Groundwater Recession Coefficient (gRC)

To classify the responsiveness of river catchments (rate of release of groundwater storage to streams^65^, e.g. slowly or quickly declining groundwater levels), we used the groundwater Recession Coefficient (gRC), which was simulated by the PCR-GLOBWB hydrology and water resources model coupled with a MODFLOW groundwater module (Supplementary Fig. 4a)^66^. We used the gRC from PCR-GLOBWB because LISFLOOD only simulates transient groundwater storage and not groundwater levels. Hence, we could not derive the gRC, i.e. the decline rate of the groundwater levels in dry periods. Note that we used the gridded groundwater Recession Coefficient (gRC) that was obtained from groundwater hydrographs rather than the more frequently used Recession Coefficient (RC), which is derived from recession limbs of the streamflow hydrograph^30^. Low gRC indicates slow release from the groundwater storage to the river, meaning that the catchment has a high memory (large storage) and is categorized as a slowly responding catchment. The gRC values for the 16 selected river catchments were roughly estimated by averaging the gRC values from all grid cells upstream of the river grid cell that represents the outlet (see Supplementary Fig. 4b). The PCR-GLOBWB model used the aquifer properties taken from the International Hydrogeological Map of Europe (IHME)^66^.

We would like to note that the gRC in a river grid cell represents only the groundwater response in that particular river grid cell. There is no influence of gRC values from the upstream cells in a catchment, which is required for this study to characterize memory in the whole catchment. The gRC in a grid cell is only determined by neighboring cells as inflow and outflow to and from the grid cell, respectively. Hence, to obtain the overall gRC of a catchment that will be assigned to the most-downstream river grid cell (catchment outlet), spatially averaging is required for the gRC of all grid cells (land and river cells) in the upstream catchment (note: not only river grid cells). Therefore, analyzing gRC values for all river grid cells ($\sim$ 10,106) would require: (1) catchment delineation for each of these river grid cells, (2) determination of the gRC of all grid cells in the delineated catchment area, and (3) calculation of the catchment-averaged gRC. This was beyond the scope of this this study, instead we obtained the gRC of 16 catchments across Europe to correlate these with the forecast performance (Fig. 4).

BFI and RC are commonly used to represent catchment response, whether it is fast or slow, which is associated with low or high catchment memory. Although BFI and RC are both derived from river flow hydrograph, they represent much more than just river system properties. In this study, we do not use the commonly applied RC, but the groundwater Recession Coefficient (gRC, see above), which even better represents catchment memory. We also note that indicators, such as hydrogeology (permeability and storage properties of aquifers and soils), the relative area of lakes/wetland/bogs, topography, land use that also describe catchment memory, however, are already implicitly embedded in the BFI and gRC.

### Brier Score (BS)

The performance of the drought forecasts was assessed using a commonly used metric called the Brier Score (BS)^27^. The BS has been used among many studies dealing with probabilistic forecasts^6,67,68^. The BS values for each LT is calculated as follow:

2 $$\begin{aligned} BS=\frac{1}{N}\sum _{i=1}^{N}(P_{i}-O_{i})^2, \end{aligned}$$

where *N* is the number of members in the forecasting ensemble, in this case is 25, *P* is the forecast probability of an event (*P* varies from 0 to 1), and *O* is the observed probability. The observed probability (*O*) consists of binary numbers either 0 for no drought event and 1 for drought event. For BS analysis, drought occurs if the SSI-x value falls below − 0.5 (mild drought)^6,16^. The BS value ranges from 0 to 1, with 0 being the perfect forecast and 1 being the worst forecast. In this paper, we average the BS values from every forecast initiation (on the second of each month), from January 2002 to December 2016, into BS values for each LT (LT = 1 to 7-month). An exception was made for seasonal analysis, where we averaged the BS values from forecast initiations in December, January, and February (DJF) for winter, March, April, and May (MAM) for spring, June, July, and August (JJA) for summer, and September, October, and November (SON) for autumn. Note that we used the Brier Score (BS) in this study that describes forecast performance, and not the Brier Skill Score (BSS), which labels forecast skill.

## Supplementary Information

Supplementary Information.

## Data Availability

The EFAS streamflow data are accessible under a COPERNICUS open data license (https://doi.org/10.24381/cds.e3458969). In this study, we used EFAS version 3. The SSI-1 and SSI-3 datasets are available in the 4TU data center with doi: 10.4121/17259449.v1. Other data and codes generated and/or analyzed during this study are available from the corresponding author on request.
