# Supplementary material for: Catchment memory explains hydrological drought forecast performance
Source: Sci Rep. 2022 Feb 17;12:2689. doi: 10.1038/s41598-022-06553-5 (PMC8854588; doi:10.1038/s41598-022-06553-5)
Supplement: Supplementary file 1 — Supplementary Information. [file 41598_2022_6553_MOESM1_ESM.pdf]

Supplementary information

Catchment memory explains hydrological drought forecast performance

Samuel J. Sutanto[1,2,3,*] and Henny A. J. Van Lanen[1]

[1] Hydrology and Quantitative Water Management Group, Environmental Sciences Department, Wageningen University and Research, Wageningen, the Netherlands
[2] Water Systems and Global Change Group, Environmental Sciences Department, Wageningen University and Research, Droevendaalsesteeg 3, 6708 PB, Wageningen, the Netherlands
[3] Institute for Marine and Atmospheric research Utrecht, Utrecht University, Princetonplein 5, 3584 CC, Utrecht, the Netherlands

*Corresponding author: Samuel Jonson Sutanto, Water Systems and Global Change Group, Wageningen University and Research, the Netherlands, email: samuel.sutanto@wur.nl.

The Supplementary Information comprises one Supplementary Note, seven Supplementary Figures, three Supplementary Tables, and Supplementary References.

Supplementary Note

To verify forecasted events that will occur ("yes") and will not occur ("no"), a dichotomous forecast method is used in our study (Stanski et al., 1989; Wilks, 2011). This method is built by developing a contingency table that shows the frequency of "yes" and "no" forecasts and occurrences (observed). A hit is counted when both the model and observed forecasts show a drought event, respectively. A miss is counted when the model forecasts no drought event, but it did occur. A false alarm is counted when the model forecasts a drought event, but it did not occur. Lastly, correct negative is counted when the model forecasts no drought event, and it did not occur. Forecast Accuracy is calculated from the fraction of hits plus correct negative divided by the total number of forecasts, while forecast success ratio (SR) is calculated from the fraction of hits divided by hits plus false alarms. A perfect forecast has accuracy and SR values of 1.

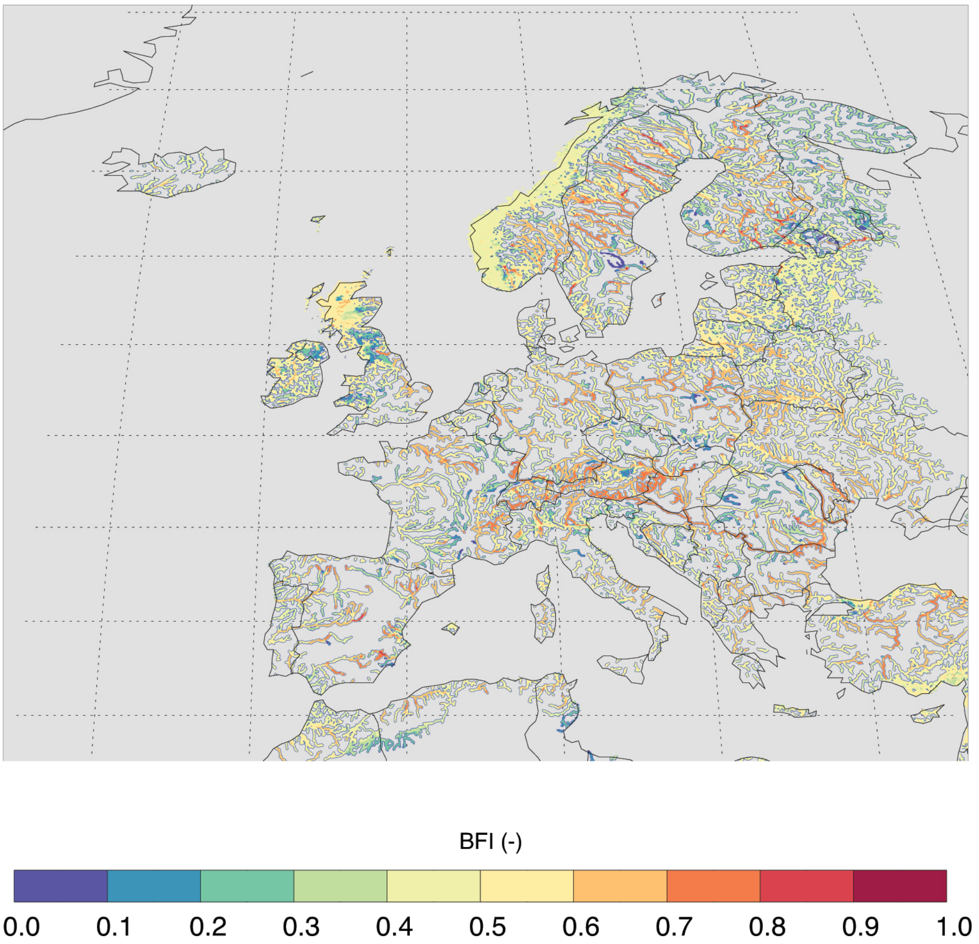

Supplementary Figure 1. The Baseflow index (BFI) for major European rivers derived from the LISFLOOD model forced with observations (SFO) from 1990 to 2018.

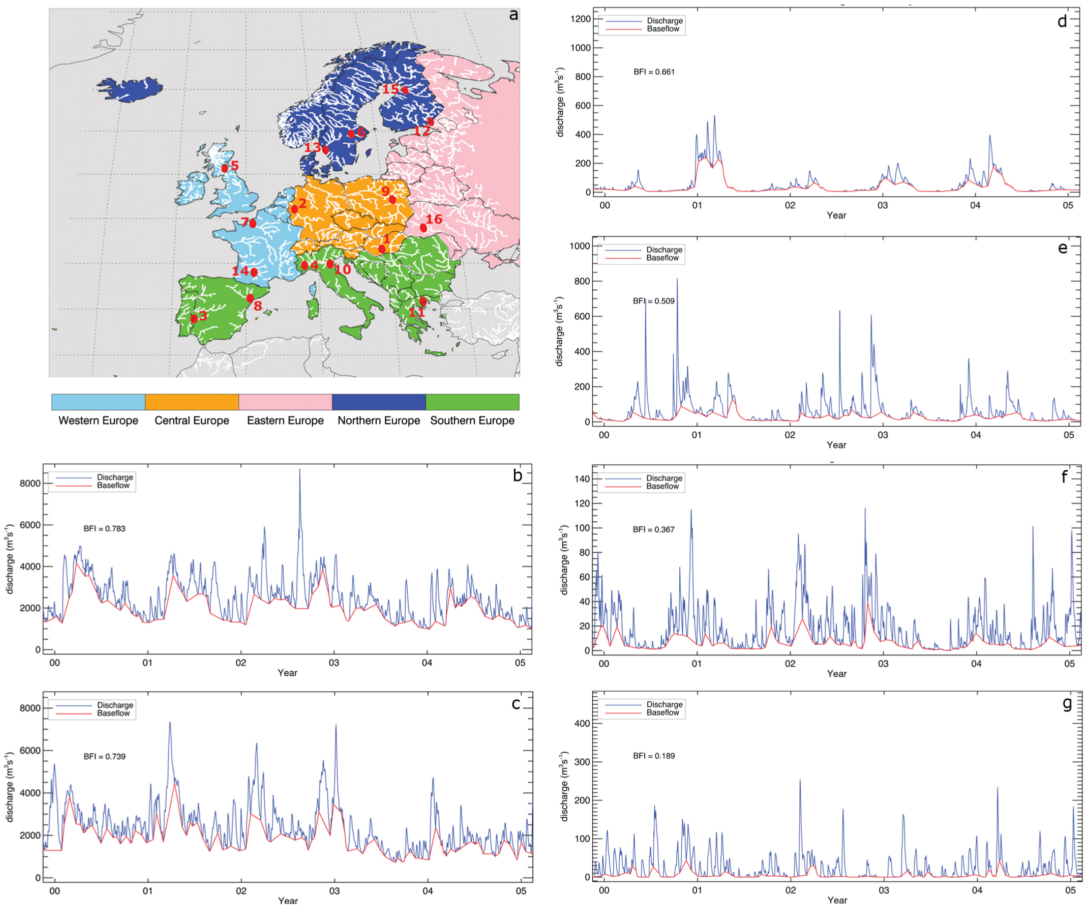

Supplementary Figure 2. (a) Map with European subregions, including the locations of 16 selected rivers: 1. Danube, 2. Rhine, 3. Guadiana, 4. Tanaro, 5. Tweed, 6. Kobacksan, 7. Seine, 8. Ebro, 9. Vistula, 10. Po, 11. Struma, 12. Vuoksi, 13. Göta, 14. Garonne, 15. Oulujoki, and 16. Dinister. The detailed Baseflow Index (BFI) analysis was only presented for six of the locations (2000-2005): (b) 1: Danube River, (c) 2: Rhine River, (d) 3: Guadiana River, (e) 4: Tanaro River, (f) 5: Tweed River, and (g) 6: Kolbacksan River. We divide European subregions into five main regions based on the United Nations geoscheme introduced by the United Nations Statistics Division (UNSD).

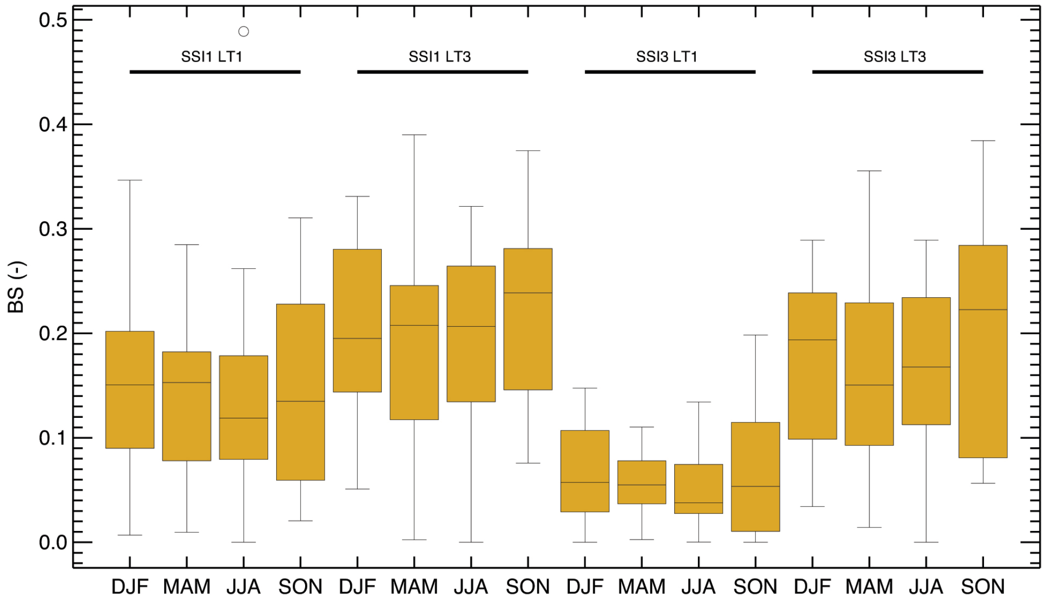

Supplementary Figure 3. Box and whisker plot showing streamflow drought forecast performance in Europe denoted by the Brier Score (BS) for SSI-1 and SSI-3 with LT = 1- and 3-month and for different seasons. Lower box shows 25 percentile, middle line shows median, and upper box shows 75 percentile. The whiskers show the 10 and 90 percentiles. Open circles indicate outliers.

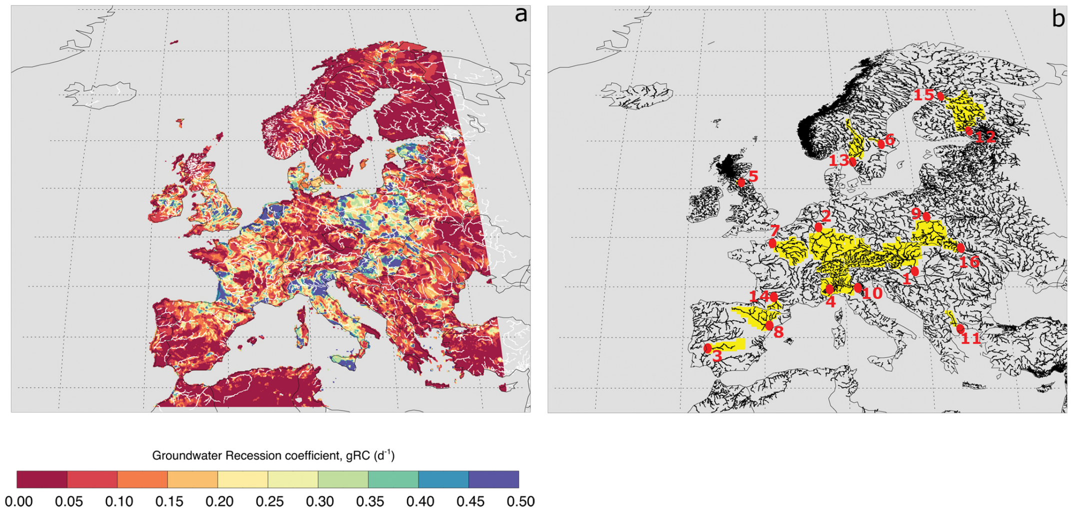

Supplementary Figure 4. (a) Groundwater Recession Coefficient (gRC) obtained from Sutanudjaja et al. (2011), and (b) locations of selected catchments in Europe with the catchment area.

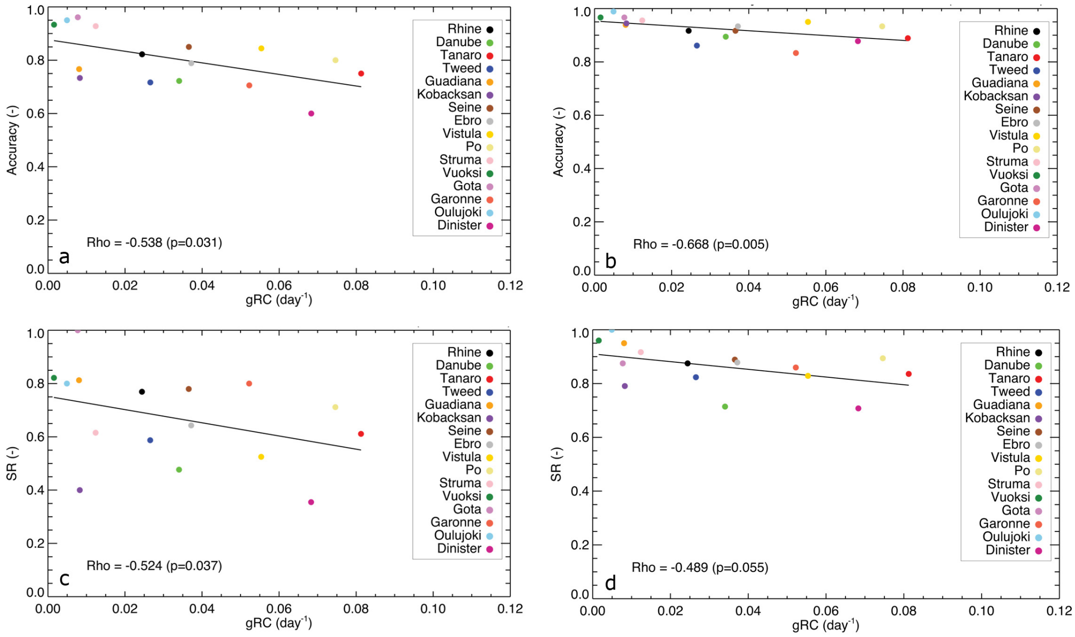

Supplementary Figure 5. Correlation between forecast performance and gRC for the 16 selected river catchments: (a) based on forecast accuracy for SSI-1 LT=1, (b) based on forecast accuracy for SSI-3 LT=1, (c) based on forecast SR for SSI-1 LT=1, and (d) based on forecast SR for SSI-3 LT=1.

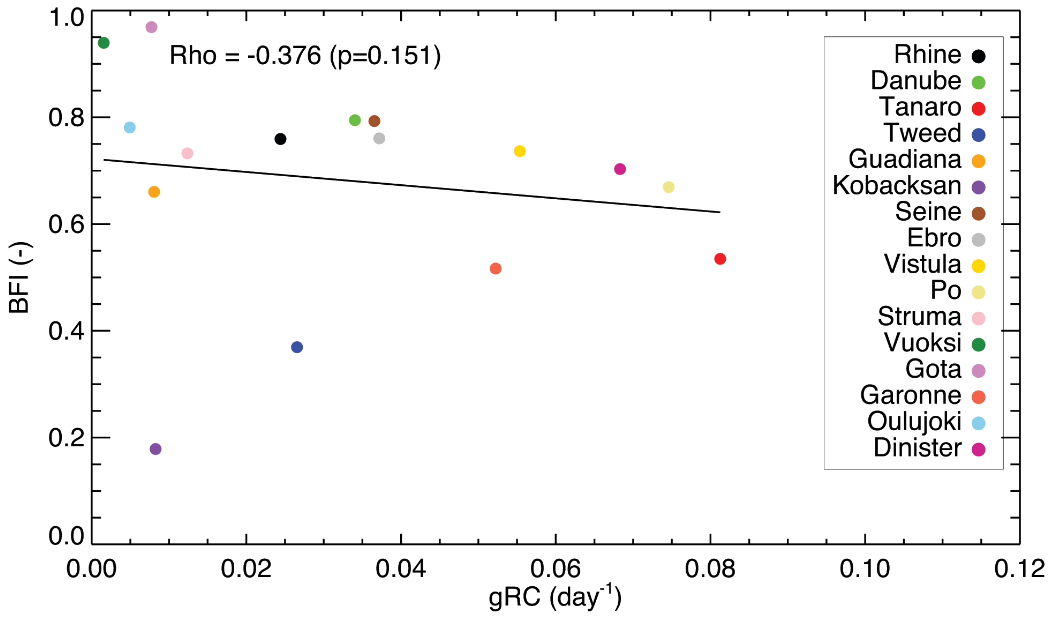

Supplementary Figure 6. Correlation of gRC and BFI for 16 selected river catchments across Europe.

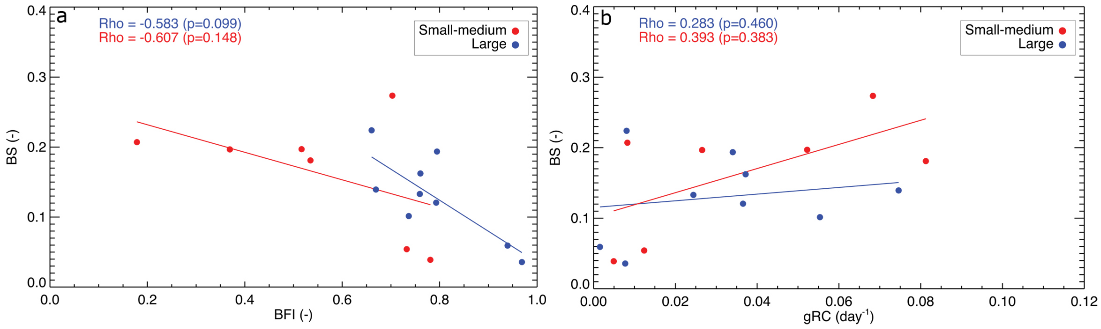

Supplementary Figure 7. Correlation between forecast performance based on forecast accuracy (BS) for SSI-1 and LT=1 for the 16 selected river catchments: (a) BFI, and (b) gRC.

We grouped Tanaro, Tweed, Kobacksan, Struma, Garonne, Oulujoki, and Dinister catchments as small to medium-sized catchments. For large catchments, we grouped Rhine, Danube, Guadiana, Seine, Ebro, Vistula, Po, Vuoksi, and Göta as large catchments.

Supplementary Table 1. Catchment characteristics for the 16 selected river catchments. P stands for precipitation, ET stands for potential evapotranspiration, R cof stands for runoff coefficient, Elevation is representative for the outlet, gRC stands for groundwater recession coefficient, and A stands for area.

No	Catchment	P (mm)	ET (mm)	R cof	Elevation (m)	gRC	A (km^2)
1	Danube	887.8	673.5	0.45	99	0.034	88,450
2	Rhine	888.8	669.5	0.46	37	0.024	67,050
3	Guadiana	413.3	1190.9	0.13	161	0.008	16,275
4	Tanaro	870.8	829.6	0.51	164	0.081	1,950
5	Tweed	1035.9	439.7	0.62	160	0.026	1,125
6	Kobacksan	635.3	486.9	0.40	19	0.008	2,275
7	Seine	701.0	702.8	0.35	2	0.036	33,375
8	Ebro	563.3	900.7	0.37	27	0.037	30,675
9	Vistula	666.1	644.9	0.27	81	0.055	40,400
10	Po	982.2	808.7	0.59	7	0.075	32,450
11	Struma	586.6	866.5	0.28	4	0.012	5,400
12	Vuoksi	624.2	426.0	0.54	39	0.002	40,825
13	Gota	685.3	484.0	0.53	3	0.008	22,625
14	Garonne	800.5	793.3	0.44	68	0.052	5,425
15	Oulujoki	598.2	390.7	0.52	18	0.005	17,100
16	Dinister	783.9	630.8	0.41	201	0.068	9,050

Supplementary Table 2. Average BFI of all river grid cells for each European subregion, and for all river grid cells in Europe. The same for the average BS and correlation coefficient (Corr) for SSI-1 and SSI-3 with lead times of 1 and 3-month. Blue color indicates the European subregion with the highest performance (low BS) and BFI for all SSIs. Red color indicates the lowest performance (high BS) and BFI. Asterix symbol means that the correlation is not significant at p = 0.01.

Drought Index	European subregions										Whole Europe	
	West Europe		Central Europe		East Europe		North Europe		South Europe			
	BFI = 0.61		BFI = 0.71		BFI = 0.64		BFI = 0.67		BFI = 0.68		BFI = 0.66	
	BS	Corr	BS	Corr	BS	Corr	BS	Corr	BS	Corr	BS	Corr
SSI-1, LT=1	0.17	-0.34	0.18	0.06*	0.19	-0.05	0.20	-0.33	0.16	-0.32	0.18	-0.21
SSI-1, LT=3	0.26	0.08	0.24	0.23	0.26	-0.06	0.28	-0.26	0.22	-0.19	0.26	-0.14
SSI-3, LT=1	0.08	-0.46	0.07	0.06*	0.08	-0.13	0.09	-0.35	0.07	-0.34	0.08	-0.26
SSI-3, LT=3	0.23	-0.14	0.22	0.19	0.23	-0.07	0.25	-0.30	0.19	-0.24	0.23	-0.18

Supplementary Table 3. gRC values for all grid cells in each of the European subregions, and all grid cells in Europe. Blue and red colors indicate the lowest and highest RC, respectively in European subregions

Subregions	gRC average
West Europe	0.038
Central Europe	0.041
East Europe	0.018
North Europe	0.012
South Europe	0.029
Whole Europe	0.027

References

1. Stanski, H.R., Wilson, L. J. & Burrows, W. R. Survey of common verification methods in meteorology. World Weather Watch Tech. Rep. No.8, WMO/TD No.358, WMO, Geneva, 114 pp. https://www.cawcr.gov.au/projects/verification/Stanski_et_al/Stanski_et_al.html. Accessed on 2021-12-01 (1989).
2. Wilks, D. S. Statistical Methods in the Atmospheric Sciences. 3rd Edition. Elsevier, 676 pp (2011).
3. UNSD. Methodology: Standard country or are codes for statistical use (M49). https://unstats.un.org/unsd/methodology/m49/. Accessed on 2020-11-17.
4. Sutanudjaja, E. H., Van Beek, L. P. H., de Jong, S. M., Van Geer, F. C. & Bierkens, M. F. P. Large-scale groundwater modeling using global datasets: a test case for the Rhine-Meuse basin. *Hydrol. Earth Syst. Sci.* **15**, 2913–2935, doi:10.5194/hess-15-2913-2011 (2011).
